# Opportunities and challenges of virtual reality-based interventions for patients with breast cancer: a systematic review

**DOI:** 10.1186/s12911-023-02108-4

**Published:** 2023-01-23

**Authors:** Alireza Banaye Yazdipour, Soheila Saeedi, Hassan Bostan, Hoorie Masoorian, Hasan Sajjadi, Marjan Ghazisaeedi

**Affiliations:** 1 Department of Health Information Management and Medical Informatics, School of Allied Medical Sciences, Tehran University of Medical Sciences, 3th Floor, No #17, Farredanesh Alley, Ghods St, Enghelab Ave, Tehran, Iran; 2 Students’ Scientific Research Center (SSRC), Tehran University of Medical Sciences, Tehran, Iran; 3 Clinical Research Development Unit of Farshchian Heart Center, Hamadan University of Medical Sciences, Hamadan, Iran; 4 Abadan University of Medical Sciences, Abadan, Iran

**Keywords:** Virtual reality, Breast cancer, Opportunities, Challenges, Systematic review

## Abstract

**Background:**

Breast cancer is one of the most common cancers diagnosed worldwide and the second leading cause of death among women. Virtual reality (VR) has many opportunities and challenges for breast cancer patients' rehabilitation and symptom management. The purpose of this systematic review is to look into the benefits and drawbacks of VR interventions for breast cancer patients.

**Methods:**

A systematic search was conducted on PubMed, Web of Science, Scopus, IEEE, and the Cochrane Library, from inception until February 6, 2022. The inclusion criteria were: (1) original studies without restriction in study design; (2) a study population consisting of patients with breast cancer; (3) any type of VR-based interventions (immersive and non-immersive); and (5) studies published in English. To assess the risk of bias, the Effective Public Health Practice Project (EPHPP) Tool was used.

**Results:**

Eighteen articles were included in this systematic review. The result showed that VR could provide many opportunities for patients with breast cancer, including reducing anxiety, time perception, pain, fatigue, chemotherapy-related symptom distress levels, and depression severity, as well as improvement in the range of motion, strength, and function. Cybersickness symptoms, the weight of headsets and helmets, the quality of the visual image, and the cost of the equipment are some of the challenges in using this technology on these patients.

**Conclusions:**

The systematic review showed that VR interventions have opportunities and challenges for patients with breast cancer. VR can be effective for rehabilitation and symptom management and is used in different stages of treatment to improve the condition of patients with breast cancer. However, before using it, the researcher should consider its challenges.

## Introduction

Breast cancer is the second leading cause of death in women and one of the most common cancers diagnosed globally [1, 2]. Globally, over one million women have been diagnosed with breast cancer annually [3]. According to GLOBOCAN 2020, there were 2.3 million newly diagnosed cases in 2020, accounting for 11.7% of all newly diagnosed cancer cases. Furthermore, breast cancer is the fifth leading cause of cancer mortality worldwide, accounting for 685,000 deaths [2].

The main types of breast cancer treatment are surgery, radiation therapy (RT), chemotherapy (CT), endocrine therapy (ET), and targeted therapy [4]. Many breast cancer survivors experience physical and psychological symptoms (such as pain, fatigue, depression, anxiety, and lymphedema); functional deficits (such as reduced shoulder range of motion and cognitive impairment); emotional problems (such as fatigue, pain, anxiety, and depression); and other complications such as bleeding, effusion, and flap necrosis. Side effects from breast cancer or treatment can have a significant impact on the quality of life (QoL) of breast cancer survivors [5–9].

With recent technological advances, the development and application of modern technology in the healthcare field offer new non-invasive approaches to managing cancer-related symptoms, and their use brings new significant benefits [10, 11]. Virtual reality (VR) technology is a distraction method defined as a noninvasive simulation technology generated in a computer-generated image or environment with width, height, and depth dimensions. This technology allows users to interact with the virtual world [12]. Current VR systems include head-mounted devices (HMDs) with stereoscopic capabilities and additional devices such as body tracking sensors, headphones, and other input hardware such as data gloves and joysticks [13].

VR can be classified as immersive, semi-immersive, or non-immersive due to its sense of presence and level of immersion. Immersive is obtained using an HMD that blocks the view of the external environment and allows the user to immerse in a three-dimensional virtual environment. In non-immersive VR, subjects interact with a scenario displayed on a screen (computer, mobile, tablet, TV) or a wall in front of a person but do not become fully immersed because they can perceive the real world together with digital images. A semi-immersive experience is something in between immersive and non-immersive VR. It takes the subjects to a partially immersive scenario displayed on a screen, and frequently they can interact with the digital scene through body movements [14, 15].

In recent years, VR has become popular in clinical research studies and used in the cancer field [7, 16, 17]. VR is a distraction intervention that can relieve symptoms such as pain, stress, anxiety, depression, fatigue, nausea, and others [18]. Most studies have shown that VR can play an essential role in patients’ empowerment and education, rehabilitation, management of cancer-related symptoms, psychiatric disorders, and side effects from treatment [7, 9, 11, 19–21]. However, this technology has drawbacks such as cybersickness, discomfort, user resistance, equipment cost, and others [22–24].

Although systematic reviews have been conducted to examine the effectiveness of VR-based interventions in the rehabilitation management of patients with breast cancer [9, 25, 26], our systematic review compared them and found some differences. In this study, we focused on both rehabilitation and symptom management of patients with breast cancer. We examined all the opportunities and benefits of using VR technology, from the mental and physical aspects it can have on breast cancer patients. Furthermore, we investigated the challenges of using this technology as well as the limitations of previous studies in this field that were not mentioned in previous reviews.

Therefore, the present study was conducted to answer the following questions: (1) What are the opportunities for VR interventions for patients with breast cancer? (2) What are the challenges, limitations and obstacles of VR interventions for patients with breast cancer? (3) What is the type of VR application (immersive or non-immersive) for patients with breast cancer? (4) In which stage of treatment was VR used? (5) What are the outcomes of using VR in breast cancer?

## Methods

### Overview

The current systematic review followed the Preferred Reporting Items on Systematic Reviews and Meta-analysis (PRISMA) guidelines [27].

### Search strategy

A systematic search was conducted using the following databases: Medline (through PubMed), Web of Science, Scopus, IEEE, and the Cochrane Library. These databases were searched from inception to 6 February 2022 for select relevant articles. Medical Subject Headings (MeSH) were used to determine the keywords. The keywords used for the search included “virtual reality”, and “breast cancer”. Mesh terms and related keywords are presented in Table 1. We reviewed the reference list of included articles to identify articles missed in the database search.

**Table 1:** Search strategy for PubMed database

Domain	Keywords	MeSH terms
Breast Cancer	“Breast Neoplasm” OR “Neoplasm, Breast” OR “Breast Tumors” OR “Breast Tumor” OR “Tumor, Breast” OR “Tumors, Breast” OR “Neoplasms, Breast” OR “Breast Cancer” OR “Cancer, Breast” OR “Mammary Cancer” OR “Cancer, Mammary” OR “Cancers, Mammary” OR “Mammary Cancers” OR “Malignant Neoplasm of Breast” OR “Breast Malignant Neoplasm” OR “Breast Malignant Neoplasms” OR “Malignant Tumor of Breast” OR “Breast Malignant Tumor” OR “Breast Malignant Tumors" OR “Cancer of Breast” OR “Cancer of the Breast” OR “Mammary Carcinoma, Human” OR “Carcinoma, Human Mammary” OR “Carcinomas, Human Mammary” OR “Human Mammary Carcinomas” OR “Mammary Carcinomas, Human” OR “Human Mammary Carcinoma” OR “Mammary Neoplasms, Human” OR “Human Mammary Neoplasm” OR “Human Mammary Neoplasms” OR “Neoplasm, Human Mammary” OR “Neoplasms, Human Mammary” OR “Mammary Neoplasm, Human” OR “Breast Carcinoma” OR “Breast Carcinomas” OR “Carcinoma, Breast” OR “Carcinomas, Breast”)	Breast Neoplasms
Virtual Reality	“Reality, Virtual” OR “Virtual Reality, Educational” OR “Educational Virtual Realities” OR “Educational Virtual Reality” OR “Reality, Educational Virtual” OR “Virtual Realities, Educational” OR “Virtual Reality, Instructional” OR “Instructional Virtual Realities” OR “Instructional Virtual Reality” OR “Realities, Instructional Virtual” OR “Reality, Instructional Virtual” OR “Virtual Realities, Instructional”)	Virtual Reality

### Selection criteria

Based on the following inclusion and exclusion criteria, a decision was made regarding including studies in this systematic review:

The inclusion criteria were (1) original studies without restriction in study design, (2) study population consisting of patients with breast cancer, (3) studies published in English language, (4) any type of VR technology (immersive or non-immersive).

Exclusion criteria were (1) reviews, meta-analyses, conference abstract, commentaries, editorials, protocols, expert opinions, and letter to editor, (2) full text not published in English, (3) unavailability of full text for data extraction, (4) studies unrelated to the purpose of the research, (5) duplicate studies, and (6) used any interventions rather than VR.

### Study selection

All studies identified were imported into EndNote X9 citation management software (Thomson Reuters, Toronto, Ontario, Canada). After removing duplicates, three authors (Alireza Banaye Yazdipour (ABY), Soheila Saeedi (SS), and Hassan Bostan (HB)) independently screened the titles and abstracts of all studies identified by the search criteria. Full texts of the remaining relevant studies were obtained, and three authors (ABY, SS, and HB) read the full-text papers and made a final selection of relevant studies. Reference lists were screened for additional eligible studies. Any disagreements were resolved by discussion and consensus between the authors and Marjan Ghazisaeedi (MG). Full-text of reviewed articles that did not meet inclusion criteria were removed, and reasons for exclusion were noted.

### Data extraction

Three reviewers performed data extraction independently (ABY, SS, and HB) using a designed form in Microsoft Excel. Any disagreement was resolved through discussion with MG. The extracted data consisted of the first author, publication year, journal or conference name, country, study design, platform, aim, type of VR application, sample size, sample description, session details, stage of treatment, type of VR technology, challenges of using VR, opportunities of using VR, limitations of the study, and outcomes.

### Quality assessment

The quality of the included studies was assessed using the “Quality Assessment Tool for Quantitative Studies” developed by the Effective Public Health Practice Project (EPHPP) [28]. This tool contains six components: (1) selection bias; (2) study design; (3) control for confounders; (4) blinding of participants and study staff; (5) validity and reliability of the data collection tools, and 6) withdrawals and drop-outs. Each component was rated as “weak”, “moderate”, or “strong” based on standardized criteria. A global rating for each study is calculated as: ‘strong’ = no weak subscale ratings; ‘moderate’ = one weak subscale rating; ‘weak’ = two or more weak subscale ratings. Each study that met inclusion criteria was assessed independently by three researchers (ABY, SS, HB). Any discrepancies were resolved by discussion and consensus between the authors and MG.

### Data analysis

The results of this study were reported descriptively, and due to the diverse outcomes and results, no meta-analysis was performed. We categorized studies that used HMD for VR intervention as immersive, while studies that didn’t use HMD were non-immersive. We categorized the limitations of the reviewed studies into two general categories: limitations related to VR technology and limitations related to the type of studies. We also divided the study's opportunities into three broad categories: no effect, positive effect, and negative effect. For studies in which statistical analysis was performed, we considered the statistically significant outcome in the intervention group as a positive effect. Furthermore, in studies in which statistical analysis was not performed, we considered the outcome that increased in the intervention group compared to the control group to be a positive effect. The authors of this study analyzed these outcomes based on deductions from the results and discussion of included studies. The VOSviewer software (version1.6.18, www.vosviewer.com) was used to identify the occurrence of keywords.

## Results

### Search output

**Fig. 1:**
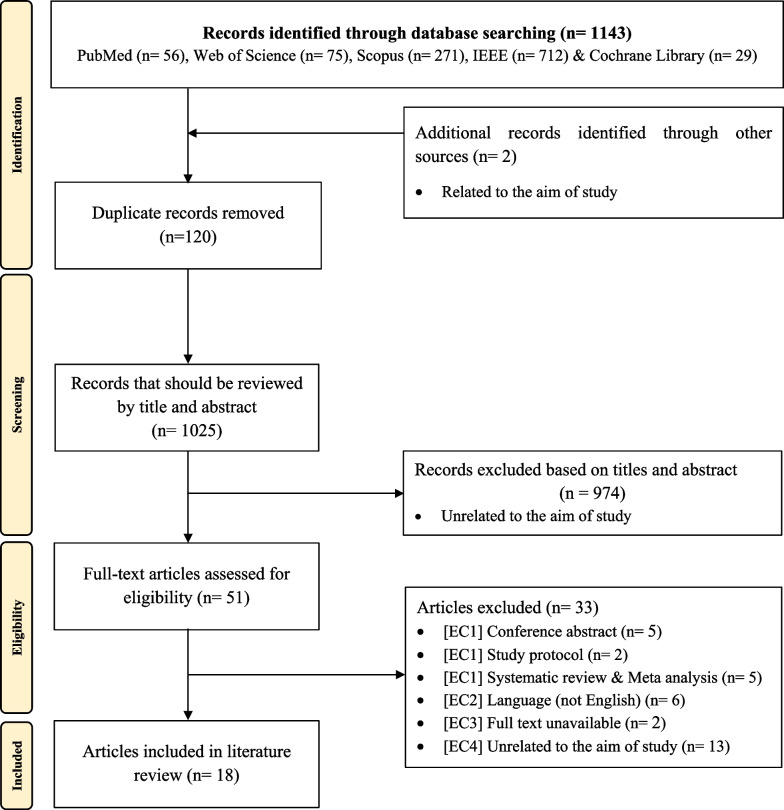
PRISMA flow diagram indicating results of identification and screening process for included and excluded papers

A total of 1143 potentially relevant articles were initially identified from the five databases; 120 articles were removed due to duplication, and the remaining 1023 studies were screened. We excluded 974 articles due to low relevance based on the title and abstract, and 49 full-text articles were screened. The characteristics of the excluded studies are shown in the PRISMA diagram. After all the eligibility criteria were applied, 16 articles were included. Two additional articles were identified by manually searching the reference lists of included articles. These two studies met our inclusion criteria. Finally, eighteen articles were included in the systematic review (Fig. 1).

### Characteristics of the included studies

The characteristics of the 18 studies are shown in Table 2. The oldest and newest studies were published in 2003 and 2021, respectively. Study designs in most of the studies were before-after (N = 5) (19, 29–32), cross-over (N = 5) [33–37], and RCT (N = 4) [7, 20, 38, 39]. Other study designs in reviewed articles were cross-sectional [40], experimental design [41], quasi-experimental [42] and quasi-randomized clinical trials [43], each with one study. All participants were adult patients with breast cancer. The type of VR technology in 9 studies (50%) mentioned that was immersive. The minimum sample size of patients was 2, and the maximum sample size of patients was 137 (IQR1: 16, median: 36.5, IQR3: 52). The intervention duration varied from 7 to 90 min.

**Table 2:** Characteristics of the 18 studies

Auther, Year, References	Country	Study design	Platform*	Aim of the study	Type of VR application	Sample size	Sample age	Intervention duration	Stage of treatment	Type of VR technology	Outcome
Schneider et al. [33]	USA	Cross-over	Computer-based with HMD	Investigation of the effect of VR on reducing chemotherapy-related symptom distress levels	Symptom distress levels reduction	N = 16	Mean ± SD: 57.7 ± 6.8	Once, mean length of time 78 min for each patient	During chemotherapy	Immersive	VR can decrease situational anxiety related to chemotherapy treatment
Schneider et al. [34]	USA	Cross-over	Computer-based with HMD	Investigation of the effect of VR on reducing chemotherapy-related symptom distress levels	Symptom distress levels reduction	N = 20	Mean ± SD: 42.6 ± 7.9	Once, mean length of time 67 min for each patient	During chemotherapy	Immersive	The distraction intervention decreased symptom distress and was well received
Schneider et al. [35]	USA	Cross-over	Computer-based with HMD	Investigation of the effect of VR on reducing chemotherapy-related symptom distress levels	Symptom distress levels reduction	N = 123	Mean ± SD: 53.97 ± 10.89	Once, average chemotherapy treatment lasted 58 min	During chemotherapy	Immersive	VR made the treatment seem shorter than treatments without distraction intervention
Schneider et al. [36]	USA	Cross-over	Computer-based with HMD	Exploration of the influence of age, gender, state anxiety, fatigue, and diagnosis on time perception with a VR distraction and predict the effects of these variables on the difference between the two groups	Time perception reduction	N = 137	Mean ± SD: 52.4 ± 10.8	Once	During chemotherapy	Immersive	Women with breast cancer are more likely to experience altered time perception during VR
Camargo et al. [29]	Brazil	Before- after	Game-based with TV screen	Investigation the applicability of VR for the recovery and rehabilitation	Rehabilitation	N = 2	Not mentioned	30 min	After surgery	Non-Immersive	VR is a valuable tool for treating secondary pain in breast cancer
House et al. [30]	USA	Before- after	Game-based with TV screen	Exploration the feasibility of VR for coping with post-surgical chronic pain and associated disability in patients	Rehabilitation	N = 6	Mean ± SD: 57.8 ± 20.4	20–50 min per session and two times a week for 8 weeks	After surgery	Non-Immersive	The findings indicate improved cognition, shoulder range, strength, function, and reduced depression
Chirico et al. [39]	Italy	RCT	Computer-based with HMD	Evaluation the effect of VR on the time perception	Time perception reduction	N = 47	Not mentioned	20 min	During chemotherapy	Immersive	Patients treated with VR underestimate the time they spend with the equipment
Bani Mohammad et al. [20]	Jordan	RCT	Computer-based without HMD	Assessment the effectiveness of VR in reducing pain and anxiety	Pain and anxiety reduction	N = 80	Mean ± SD: 51.99 ± 10.34	once	Patients during the care process	Non-immersive	VR is an effective distraction intervention for managing pain and anxiety among breast cancer patients
Jimenez et al. [42]	Australia	Quasi-experimental	Computer-based without HMD	Investigation the impact of Virtual Environment for Radiotherapy Training on patients’ RT knowledge and anxiety	Knowledge and positive experience enhancement	N = 37	Range: 35–74	“8 months 1st of April 2015 and 27th of November 2015”	During radiation therapy	Non-Immersive	This examination reports the significance of education programs in enhancing radiation therapy knowledge and perhaps decreasing patient anxiety
Chirico et al. [7]	Italy	RCT	Computer-based without HMD	Assessment of the efficacy of VR in relieving chemotherapy‐related anxiety and negative mood states	Anxiety reduction and mood states improvement	N = 94	Mean ± SD: 55.18 ± 5.7	45–90 min	During chemotherapy	Non-Immersive	VR and MT are helpful interventions for improving mood states and relieving anxiety, depression, and fatigue during chemotherapy
Pizzoli et al. [31]	Italy	Before- after	Mobile-based with HMD	Comparison of relaxation techniques in VR	Relaxation	N = 16	Mean ± SD: 47.7 ± 7.24	7 min	Patients during the care process	Immersive	Initial outcomes indicate that using body-focused exercises for patients might cause major efficacy compared to breathing exercises that have been broadly utilized until now in relaxing VR
Feyzioğlu et al. [38]	Turkey	RCT	Game-based with Xbox Kinect	Investigation the effects of VR on pain, ROM, muscle strength, functionality, and fear of movement	Rehabilitation	N = 40	Mean ± SD: 50.84 ± 8.53	"11 months Both groups received the treatment for 45 min per session and two times a week for 6 weeks."	After surgery	Non-Immersive	VR training using Xbox Kinect™ might be as effective as standard physiotherapy in the management of upper limb dysfunctions
Durosini et al. [40]	Italy	Cross-sectional	Not mentioned	Exploration BC survivors’ attitudes towards internet-based psychotherapy	Attitudes investigation	N = 48	Mean ± SD: 50.23 ± 7.06	Not mentioned	Patients with history of Breast cancer	Not Mentioned	BC survivors tend to perceive Videoconferencing and VR as the most useful, effective, reassuring, and reliable modalities
Piejko et al. [32]	Poland	Before- after	Computer-based without HMD	Understanding the impact of VR to improve postural control	Rehabilitation	N = 46	Mean ± SD: 51.67 ± 6.62	6 days a week (from Monday to Saturday), for 45 min a day	After surgery	Non-immersive	Treatment did not affect static postural control but contributed to the improvement of dynamic postural control
Atef et al. [43]	Egypt	Quasi-randomized clinical trial	Game-based with TV screen	Identifying and comparing the therapeutic advantages of VR-based exercises for postmastectomy lymphedema	Lymphedema reduction and rehabilitation	N = 30	Mean ± SD: 54.07 ± 8.28 and 53.07 ± 7.24 years for the VR and PNF groups	2 sessions per week for 4 weeks [8 sessions]	After surgery	Non-immersive	VR is beneficial in reducing postmastectomy lymphedema
Zhou et al. [41]	China	Experimental design	Computer-based with HMD	Developing a VR upper limb rehabilitation system for patients and explore its usability	Rehabilitation	N = 15	Mean ± SD: 54.73 ± 7.78	15 days	After surgery	Immersive	VR rehabilitation system is feasible and easy to learn
Buche et al. [19]	France	Before- after	Game-based with HMD	Examination the benefits of VR using two immersion methods and comparing them with each other	Rehabilitation	N = 52	Mean ± SD: 56.02 ± 10.62	"10 months Each session lasted an average of 30 min."	After surgery	Immersive	The therapeutic benefits of VR are mainly associated with its distractive power
Ashley Verzwyvelt et al. [37]	USA	Cross-over	Computer-based with HMD	Investigation the effects of biophilic green therapy or VR environment on decreasing pain and distress	Pain and distress reduction	N = 33	Mean ± SD: 59.03 ± 13.2	Not mentioned	During chemotherapy	Immersive	The engagement of nature eased some of the burden experienced during treatment and encouraged patients to further explore its benefits

^*^Platform: The hardware that leads to a person's communication with VR

*VR* virtual reality, *HMD* head-mounted display, *RT* radiation therapy, *MT* music therapy, *ROM* range of motion, *BC* breast cancer, *PNF* Proprioceptive neuromuscular facilitation

**Fig. 2:**
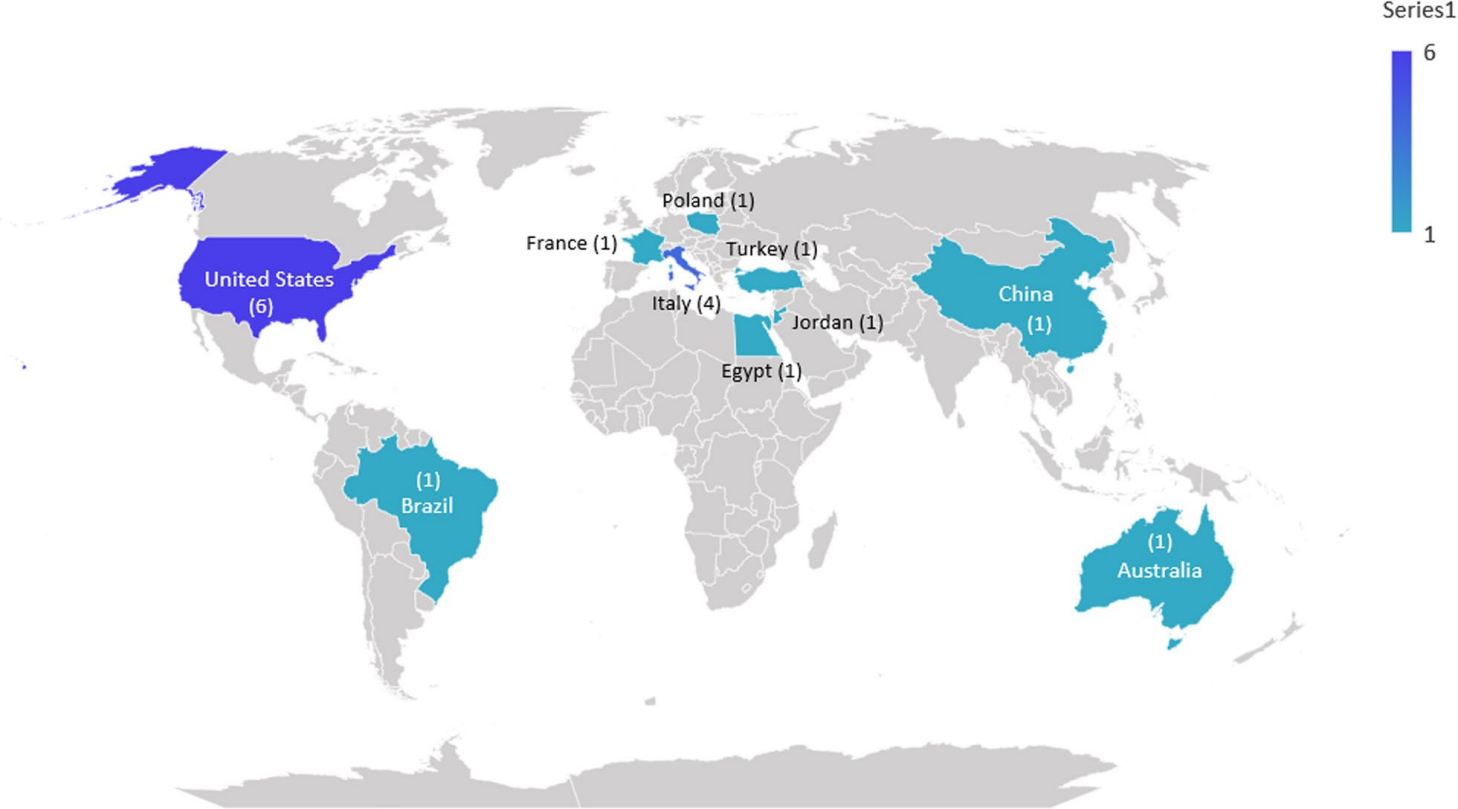
The distribution of studies based on their conducted countries

Figure 2 shows six studies from the United States [30, 33–37], four from Italy [7, 31, 40, 44], and one from Australia [42], Brazil [29], China [41], Egypt [43], France [19], Jordan [20], Poland [32], and Turkey [38].

**Fig. 3:**
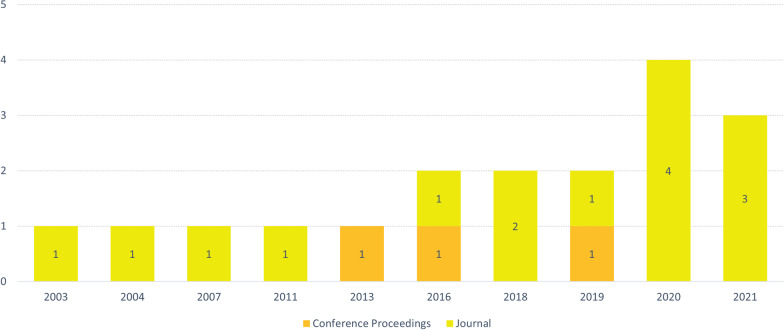
The distribution of studies based on publication year and type

Figure 3 shows that most studies are published in journals (15 of 18 studies; 83.3%) and between 2016 to 2021 (13 of 18; 72.2%).

**Fig. 4:**
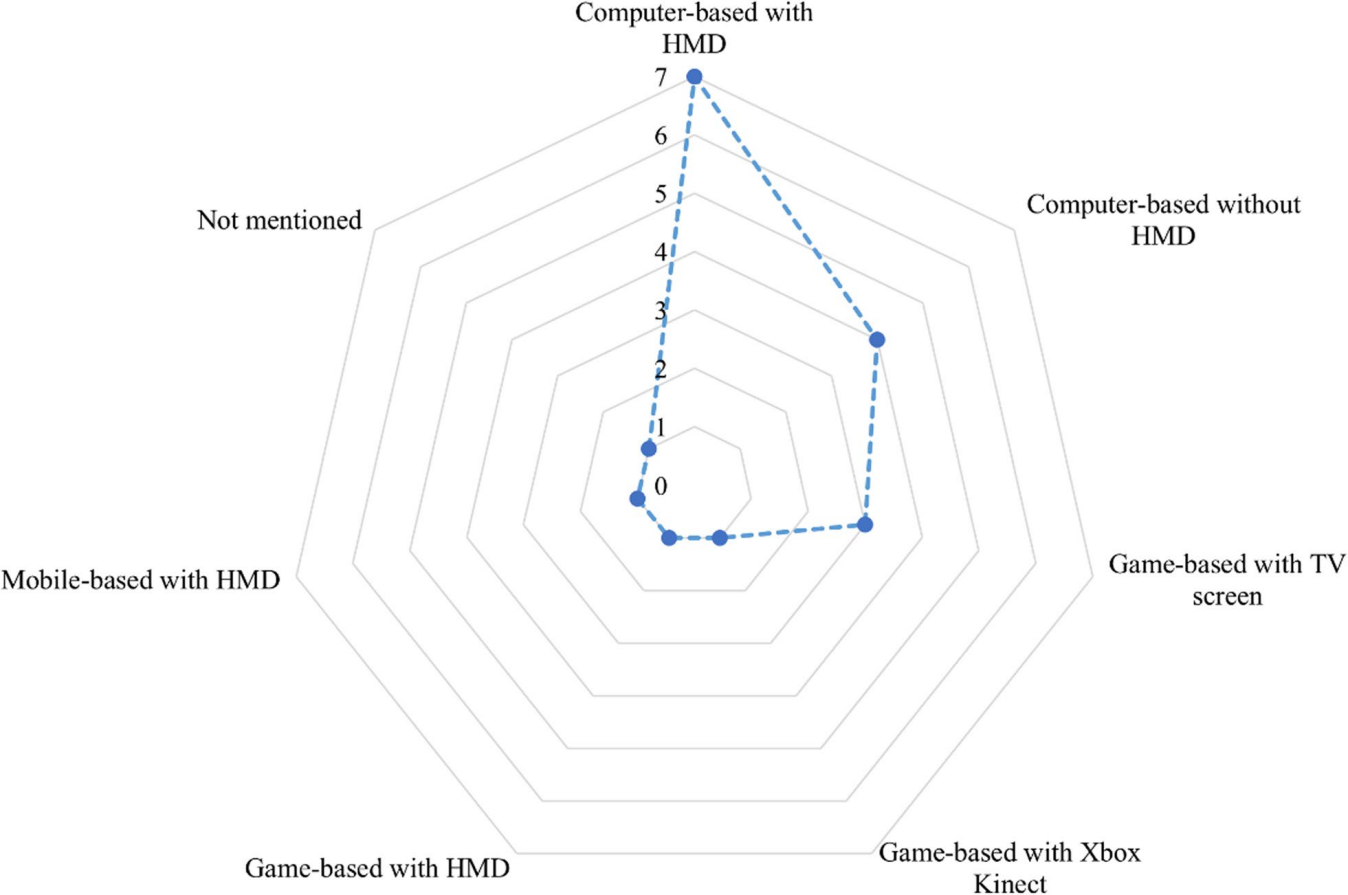
The radar chart of the platforms used for VR intervention

Figure 4 shows that most VR intervention platforms were computer-based with HMD (7 of 18 studies; 38.8%). In 4 of 18 studies (22.2%), VR intervention platforms were computer-based without HMD.

**Fig. 5:**
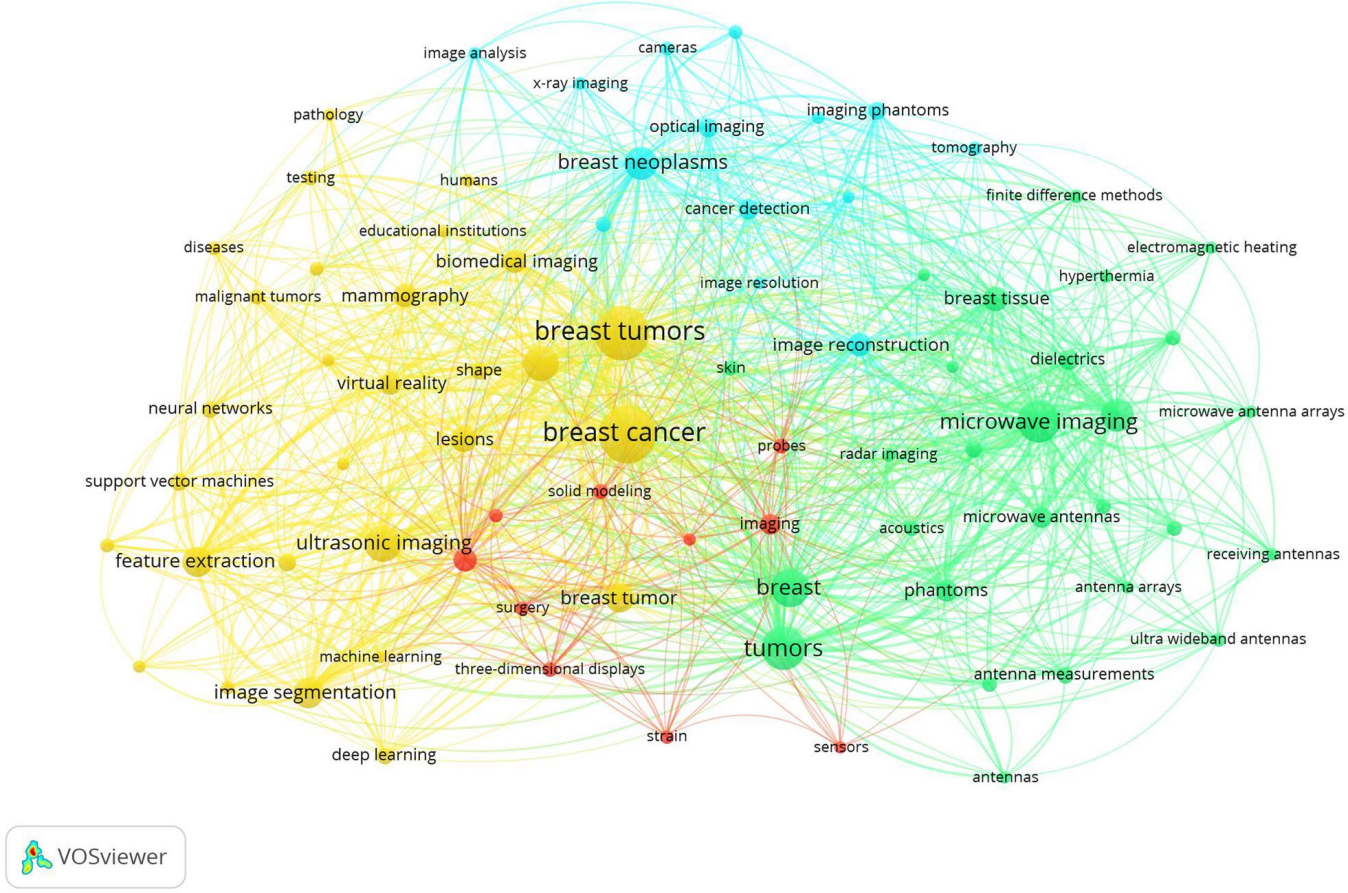
Co-occurrence overlay visualization network of keywords

Figure 5 shows VOSviewer's overlay visualization of keyword co-occurrence. Each node in the network represents a keyword, and the size of the circle indicates the occurrence frequency. The distance between these keywords on the VOSviewer map reveals the relationship between these keywords. The more the two keywords appear together, the closer they are to the network. Larger circles representing keywords such as breast cancer, breast tumors, and tumors indicated that these keywords appeared more frequently. VR is closer to breast cancer and breast tumors, which indicates the importance of VR interventions in this field.

### Challenges and limitations of included studies

The challenges and limitations of included studies were classified into two categories: VR and study-related (Table 3).

**Table 3:** Challenges and limitations of included studies

Challenges categories	Challenge/ Limitation/ Obstacle subcategories	Studies
VR-related	Weight of headsets and helmets	[33, 41]
	User resistance because of first exposure to the VR	[19, 41]
	Quality of visual image	[33]
	Weakness of learning ability	[41]
	Need for familiarization stage to use of VR	[19]
	Adverse effects of medication on the effective use of VR	[37]
	Cost of the equipment	[20]
Study-related	Small sample size	[30, 33, 34, 37, 41–43]
	Study design	[7, 31, 32, 36, 38, 42]
	Single study site	[33–35, 42]
	Lack of generalizability	[20, 41, 42]
	Risk of bias	[7, 36, 42]
	Lack of standardized measurement tools	[30, 35]
	The intervention was used only once with each patient	[7, 35]
	Lack of blinding	[7]
	Investigating short‐term effects	[7]
	Short duration of the study	[38]
	Non-compliance of patients	[43]
	Insufficient data collection	[41]
	Patient unwillingness to complete questionnaires	[19]

#### Challenges and limitations of VR-related

Most of the challenges and limitations in the VR category were 1) the weight of headsets and helmets and 2) User resistance because of first exposure to the VR.

#### Challenges and limitations of study-related

In the study category, most of the challenges and limitations were 1) small sample size, 2) study design, 3) single study site, 4) lack of generalizability, and 5) risk of bias.

### Outcomes and opportunities of VR intervention

Table 4 presents the outcomes and opportunities of VR intervention in breast cancer use. The outcomes and opportunities of VR in included studies are classified into two categories: mental and physical.

**Table 4:** Outcomes of VR intervention in included studies

Outcome category	Outcomes subcategories	Effect		
		Positive	No effect	Negative
Mental aspects	Reducing chemotherapy-related symptom distress levels	[33, 34, 37]	[35]	
	Reducing fatigue	[7, 33, 34]		
	Reducing anxiety	[7, 19, 20, 33, 34]		
	No cybersickness	[19, 30, 31, 33, 34, 35]		[7]
	Reducing the time perception	[19, 34, 35, 36, 44]		
	Reducing pain	[20, 29, 30, 38]		[37]
	Reducing depression severity	[7, 30]		
	No motion sickness	[30]		
	Improving knowledge about treatment	[42]		
	Increase of confidence	[42]		
	Increase of satisfaction	[42]		
	Reducing tension	[7]		
	Reducing anger	[7]		
	Relaxation	[31]		
	Patients experienced a more pleasant state	[31]		
	Reducing fear of movement	[38]		
	Reducing negative emotional arousal	[19]		
	Increase in positive emotional state	[19]		
Physical aspects	Strength and function metrics improved	[30, 38, 43]		
	Increase of range of motion metrics	[30, 38]		
	Reducing heart rate	[37]		
	Reducing blood pressure	[37]		
	Reducing saliva cortisol		[37]	
	Improvement of lymphedema state	[43]		
	Static postural control		[32]	
	Dynamic postural control	[32]		
	Increase of flexion and abduction	[29]		
	Increase of electrical activity	[29]		

#### Mental outcomes

The mental aspects of outcomes related to VR intervention mentioned in the studies included reducing anxiety, time perception, pain, chemotherapy-related symptom distress levels, fatigue, depression severity, and other. The most positive mental effects were related to the three subgroups, such as reducing anxiety and time perception (five studies), reducing pain (four studies), reducing chemotherapy-related symptom distress levels, and fatigue (three studies).

#### Physical outcomes

The physical aspects of outcomes related to VR intervention mentioned in the studies included improving the strength and function metrics, increasing the range of motion metrics, reducing heart rate and blood pressure, and other. The most positive physical effects were related to the three subgroups, such as improving the strength and function metrics, reducing fatigue (three studies) and increasing the range of motion metrics (two studies).

### Quality assessment of the included studies

**Fig. 6:**
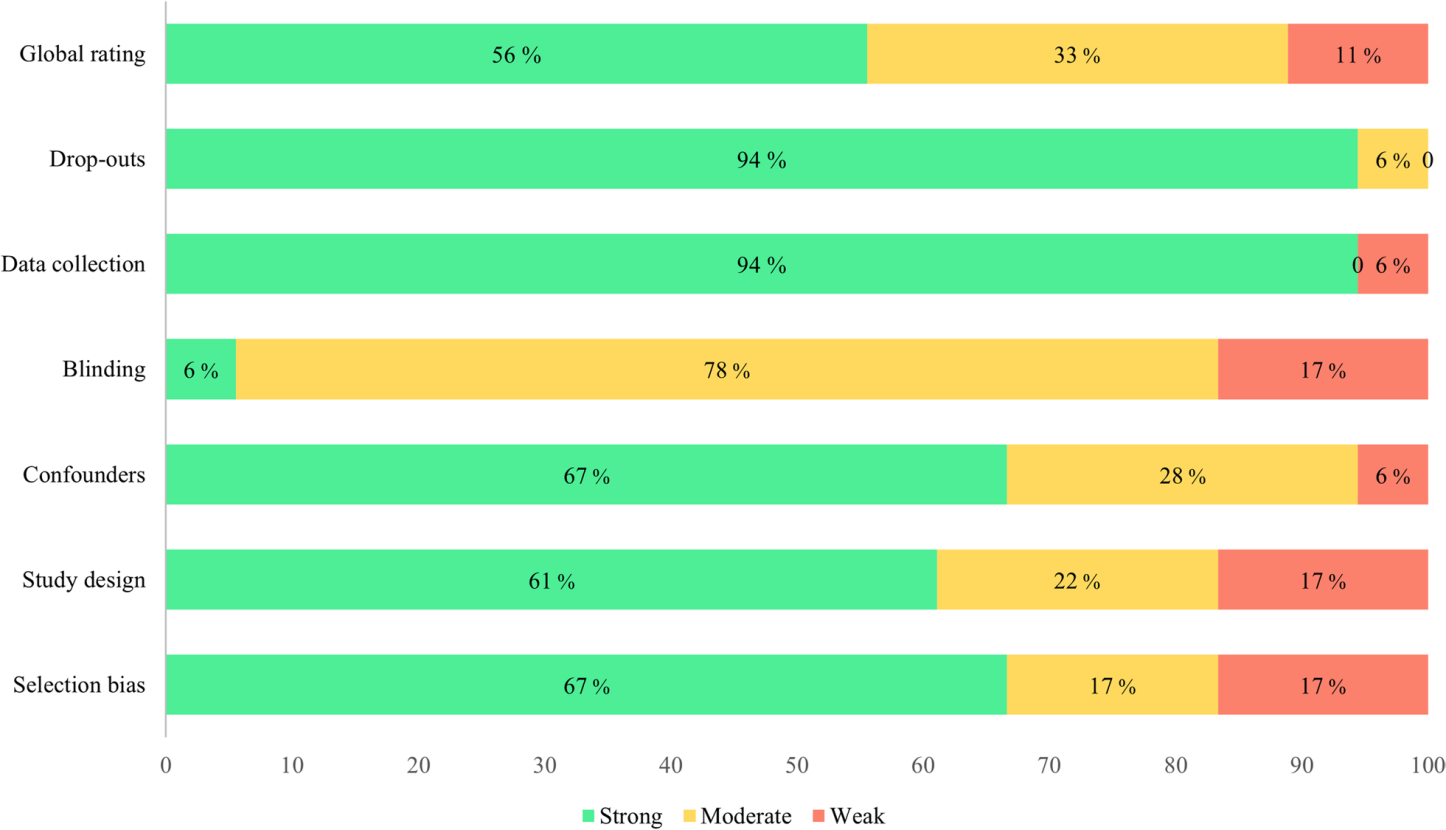
Quality assessment of the included studies

The results of the quality assessment are shown in Fig. 6. Based on the sum of scores, most studies were strong in terms of drop-outs and data collection (94%), and moderate in terms of blinding (78%). Concerning the global rating score, 56% of the included studies were strong, 33% moderate, and 11% weak.

## Discussion

This systematic review examined the opportunities and challenges of VR intervention in patients with breast cancer and discussed the opportunities, challenges, and positive and negative effects of its mental and physical aspects. The most important benefits and opportunities of using VR from a mental perspective in this group of patients were that it reduced anxiety and pain and led patients to underestimate the time spent during treatment using VR technology compared to the duration of the treatment without this technology. In a systematic review, Chow et al. found that the VR was an effective distraction for reducing pain and anxiety for patients with cancer undergoing medical interventions or receiving chemotherapy. The findings of this systematic review are congruent with previous reviews. They show that VR intervention can effectively manage mental aspects such as anxiety, time perception, pain, fatigue, and depression severity [45–48]. Based on the distraction mechanism in adult and pediatric cancer patients at various stages, the possible reason for the effectiveness of VR intervention in managing mental aspects can be considered [49–51]. Schneider et al., in their studies, found that VR can reduce anxiety in patients undergoing chemotherapy. In addition, patients undergoing chemotherapy suggested that anxiety was reduced primarily by an altered perception of time, a sense of fun and enjoyment, and astonishment that the session had been completed [34, 35]. However, potential factors that may have influenced the results must be investigated and taken into account for future research.

This technology also had several physical benefits for patients, such as improving function metrics and increasing the range of motion metrics. VR technology may increase individuals' motivation and participation in treatment programs. In addition, they are allowing a wide range of possible movements and exercises to be implemented in rehabilitation programs. A systematic review by de Araújo et al. showed that VR-based interventions in different rehabilitation protocols improved motor function, balance, aerobic function, driving skills, pain level, and psychological and motivational aspects [52]. Most studies found that VR intervention can be effective in upper limb rehabilitation and improve physical aspects such as strength and function metrics and range of motion metrics [48, 53–55], consistent with our findings. Quality of life (QoL) is related to the level of physical activity. Therefore, physical exercise programs are used to rehabilitate patients treated with chemotherapy. VR interventions could help improve the level of physical activity and QoL.

Cybersickness symptoms were not reported in most studies for using VR in breast cancer patients' treatment, which indicates that technology is advancing to the point where cybersickness symptoms are no longer evident. The findings of this systematic review are in line with previous studies [9, 48]. Cybersickness refers to symptoms and unpleasant side effects that users experience during or after VR immersion, such as nausea, headache, dizziness, vomiting, eyestrain, tiredness, disorientation, ataxia, pallor, dry mouth, and sweating [56, 57]. Chirico et al. in their study reported cybersickness symptoms using the Virtual Reality Symptom Questionnaire (VRSQ) [58]. Their findings showed that except for a slight difficulty in concentrating, all symptoms such as nausea, dizziness, headache, drowsiness, and eyestrain occurred with a frequency of less than 20% in the patients [7]. In a systematic review and meta-analysis, Caserman et al. found that advancements in technology and current-generation VR HMDs have significantly fewer problems with cybersickness (*P* < 0.001), which could be due to technological advances. However, some symptoms of cybersickness are still present. Furthermore, they discovered that the nature of movement, specifically sensory mismatch and perceived motion, were the primary causes of cybersickness in VR [57].

This systematic review indicated that VR had negative effects in only two studies, including cybersickness in one study and no reduction in pain in another. Our study revealed that the application of VR may always bring challenges. The most critical challenges reported in studies related to this technology include two challenges: (1) the weight of headsets and helmets; and (2) the resistance of patients affected by breast cancer against using VR because of their first exposure to it. User resistance is a complex behavior phenomenon that is considered as an important constraint in the successful implementation and use of technology [59]. When a new technology such as VR is used for the first time, patients may resist using it due to a lack of familiarity and fear use it [60]. However, more studies are necessary about the resistance of patients affected by breast cancer against using VR.

Safi et al. in their study found that engaging and supporting stakeholders in developing new technologies such as VR is essential and can reduce user resistance, which leads to increased technology acceptance in individuals [61]. As a result, it is suggested that patients receive the necessary education to become acquainted with emerging technologies such as VR.

This study showed that therapists could use VR in different stages of treatment to improve the condition of patients with breast cancer. As mentioned, VR can be used in chemotherapy, radiotherapy, and the post-surgery period, and therapists can achieve different goals with this technology in these stages of treatment. For example, they can be used in patients who have had a mastectomy to increase their range of motion. In the chemotherapy stage, it can also be employed to reduce time perception, which many studies have shown that it was instrumental in this goal and has led to less understanding of the time spent. On the other hand, this technology can be utilized to reduce fatigue or even depression in these patients. The findings of this systematic review are consistent with previous reviews on this topic [9, 48, 50]. VR intervention appears to be a powerful and effective tool for diverting patients' attention away from medical procedures such as chemotherapy [50].

This study had a series of strengths and limitations. One of the strengths of this study was searching five valid databases and examining the references of all the included studies, which led to including the most relevant studies in this review as much as possible. However, this study has some limitations that need to be addressed. Firstly, the number of trials was small. Secondly, the included studies involved small sample sizes, highlighting the need to develop trials with larger population sizes. Thirdly, studies in non-English languages should also be considered.

### Implication for practice and future research

The results of this systematic review demonstrated that using VR intervention in breast cancer patients decreases anxiety, pain, depression, fatigue, time perception, fear of movement, and cognition function. In addition, the technology increases relaxation, knowledge, confidence, satisfaction, strength and function metrics, and range of motion metrics. Moreover, cybersickness symptoms were rare, and this reflects the advancement of VR technology. Based on these findings, it is recommended that healthcare providers use VR intervention for patients with breast cancer during the care process, chemotherapy, radiation therapy, and after surgery. The development of VR programs that empower patients to continue their therapy at home can be helpful because the treatment does not end when the patient leaves the oncology ward. Future studies can be conducted with larger sample size, longer intervention duration, and higher methodological quality. Furthermore, it is suggested that this intervention's cybersickness symptoms and cost-effectiveness be examined.

## Conclusion

This systematic review showed that VR interventions could serve as a tool for supporting breast cancer patients. VR could provide opportunities to reduce anxiety, time perception, pain, fatigue, chemotherapy-related symptom distress levels, and depression severity and improve the range of motion, strength, and function. However, some challenges include the weight of headsets and helmets, visual image quality, and equipment cost. VR can be effective for rehabilitation and symptom management and is used in different stages of treatment to improve the condition of patients with breast cancer. However, cybersickness’s clinical factors are poorly understood and need further research.

## Data Availability

All data generated or analyzed during this study are included in this published article.

## Abbreviations

VR: Virtual Reality
QoL: Quality of Life
HMD: Head-Mounted Display
RT: Radiation Therapy
MT: Music Therapy
ROM: Range of Motion
BC: Breast Cancer
PNF: Proprioceptive Neuromuscular Facilitation
